# METTL3-Mediated N6-Methyladenosine Modification of Trim59 mRNA Protects Against Sepsis-Induced Acute Respiratory Distress Syndrome

**DOI:** 10.3389/fimmu.2022.897487

**Published:** 2022-05-25

**Authors:** Yi Chen, Yuling Wu, Linjie Zhu, Caiyang Chen, Saihong Xu, Dan Tang, Yingfu Jiao, Weifeng Yu

**Affiliations:** ^1^ Department of Anesthesiology, Renji Hospital, Jiaotong University School of Medicine, Shanghai, China; ^2^ The Cancer Hospital of the University of Chinese Academy of Sciences (Zhejiang Cancer Hospital), Institute of Basic Medicine and Cancer (IBMC), Chinese Academy of Sciences, Hangzhou, China

**Keywords:** sepsis, acute lung injury, endothelial barrier, epigenetic regulation, m^6^A, METTL3

## Abstract

N6-methyladenosine (m^6^A) RNA modification is a fundamental determinant of mRNA metabolism in eukaryotic cells and is involved in numerous physiological and pathological processes. However, the specific role of m^6^A modification in sepsis-induced acute respiratory distress syndrome(ARDS) remains unknown. Here, we show that the levels of m^6^A RNA were significantly decreased in septic lungs and that METTL3 was the main regulator involved in the absence of m^6^A RNA modification. Pulmonary endothelial barrier damage is a critical process in the pathogenesis of acute lung injury during sepsis. METTL3 regulated endothelial barrier dysfunction and inflammatory responses in sepsis-induced ARDS *in vivo* and *in vitro*. Furthermore, we identified tripartite motif-containing (Trim)59 as a key m^6^A effector and Trim59 deficiency exacerbated lung injury. Mechanistically, METTL3 inhibited endothelial injury in sepsis-induced ARDS through Trim59-associated NF-κB inactivation. Our findings revealed novel insights into epitranscriptional mechanisms in sepsis-induced ARDS *via* m^6^A modifications, which has important application value in the diagnosis, prognosis, and molecular-targeted therapy of sepsis-associated lung injury.

## 1 Introduction

Sepsis is life-threatening organ dysfunction caused by a dysregulated host response to infection (1). The lung is the most vulnerable organ in patients with severe sepsis (2). Approximately 50% of critically ill patients with severe sepsis may develop acute respiratory distress syndrome (ARDS), which has a mortality rate as high as 30-40%. To date, no specific therapy has been shown to be effective against sepsis-induced ARDS (3, 4). This condition is an urgent clinical problem, and further exploring the pathogenesis and mechanisms of sepsis-related lung injury will provide new therapeutic strategies for sepsis, thereby reducing mortality in intensive care units.

A key pathological characteristic of ARDS is the disruption of lung endothelial barrier function (5, 6). The endothelium provides a semipermeable barrier between circulating blood and tissues. In ARDS, the endothelial barrier is disrupted due to inflammatory stimulation, leading to the accumulation of protein-rich inflammatory edematous fluid, hyaline membrane formation, and pulmonary infiltration of neutrophils, resulting in poor lung compliance, diffuse alveolar damage, severe hypoxemia and even acute respiratory failure (7–9). Therefore, preserving the integrity of the vascular endothelium may help to develop potentially novel therapeutic methods for sepsis-induced ARDS.

Recently, many genetic and epigenetic changes have been shown to be involved in endothelial function maintenance (10–13). Accumulating evidence suggests that pulmonary microvascular leakage and organ injury/dysfunction associated with sepsis are associated with changes in epigenetic and gene expression (14). Chemical modifications of RNA molecules, which are known as the epitranscriptome, play an essential role in the posttranscriptional regulation of gene expression (15). It has been shown that N^6^-methyladenosine (m^6^A) affects numerous physiological and pathological processes. m^6^A modification is the most abundant form of mRNA and long noncoding RNA (lncRNA) methylation in eukaryotic cells. Increasing evidence suggests that m^6^A modification is involved in regulating RNA splicing, localization, stability, and translation. m^6^A modification is catalyzed by methyltransferases (METTL3, METTL14, etc.) and removed by demethylases (FTO, ALKBH5, etc.) to regulate the synthesis and degradation of mRNAs. YTH family proteins, IGF2BPs, and hnRNPA2/B1 have been shown to affect mRNA stability and translation (16, 17). It has been reported that abnormal m^6^A mRNA methylation is intimately involved in the onset and progression of human diseases, suggesting that m^6^A modification plays an important role in different genomic backgrounds (18). However, the role of m^6^A modification in sepsis-induced ARDS remains largely unknown. Therefore, it is necessary to elucidate the underlying mechanism to reveal the exact biological process and effects of m^6^A modification on sepsis-induced pulmonary endothelial injury.

Here, we showed that a decrease in m^6^A modifications was associated with downregulated METTL3 expression in sepsis. METTL3 knockdown accelerated barrier dysfunction and inflammatory responses. Furthermore, tripartite motif-containing (Trim59) was identified as a key m^6^A effector, the deficiency of which exacerbated lung injury. Mechanistically, the m^6^A reader YTHDF1 recognized and stabilized m^6^A-modified Trim59 mRNA to protect the vascular endothelium against barrier dysfunction and inflammatory responses. These data revealed novel insights into epitranscriptional mechanisms in sepsis-induced ARDS *via* m^6^A modification, which has important application value in the diagnosis, prognosis, and molecular-targeted therapy of sepsis-associated lung injury.

## 2 Materials and Methods

### 2.1 Animal Model

All animal experiments were approved by the Renji Hospital Institutional Animal Care and Use Committee and were performed in accordance with the Institutional Guide for the Care and Use of Laboratory Animals. Male 6- to 8-week-old C57BL/6J mice were purchased from Shanghai SLAC Laboratory(Shanghai, China). The mice were housed with a 12-h light/dark cycle in a temperature-controlled room with water and pelleted chow provided *ad libitum*.

#### 2.1.1 Delivery of the Entranster™-*In Vivo*/siRNA Complex

The mice were anesthetized with isoflurane and then intranasally administered METTL3 siRNA (20 nmol) or NC siRNA. The mixing ratio of siRNA (μg) to Entranster™-*in vivo* (μl) was 1:0.5.

#### 2.1.2 CLP Model 

CLP was performed as previously described (19). The mice were anesthetized with isoflurane and subjected to a laparotomy. The cecum was exposed, ligated with a 3/0 silk suture and punctured with a 21-gauge needle. Then, the cecum was placed back into the abdomen. Saline was injected subcutaneously immediately after surgery. Lung tissues, BALF and blood samples were collected 24 h after CLP. Animal deaths were monitored for 7 days, and the survival rates were calculated.

### 2.2 Cell Culture and Treatment

#### 2.2.1 Cell Culture

The HLVEC line (HULEC-5a) was purchased from ATCC and cultured in MCDB131 medium supplemented with 10% fetal bovine serum, 10 ng/ml EGF, 1 µg/ml hydrocortisone, 10 mM L-glutamine, 100 U/ml penicillin, and 100 µg/ml streptomycin. Mouse PMVECs were isolated from male C57BL/6 mice (2-4 weeks old). Briefly, the mice were sacrificed and perfused with PBS *via* the right ventricle to remove blood from the lungs. The lungs were removed, and peripheral tissue was cut into pieces and cultured in DMEM containing 20% fetal calf serum, 25 mM HEPES, 3.7 g/L NaHCO3, 5 mg/ml heparin, 1 mg/ml hydrocortisone, 80 mg/ml endothelial cell growth supplement from bovine brain, 5 mg/ml amphotericin, and 0.01% ampicillin/streptomycin at 37°C with 5% CO2 for 72 h. Then, the diced tissue was removed, and the adherent cells were cultured in basal culture media. PMVECs are characterized by an analogous round morphology and Von Willebrand Factor (vWF) staining, with positive staining of approximately 90% (**Supplementary Figure 6**). PMVECs passaged between three and five were used in experiments.

#### 2.2.2 Cell Treatment

For endothelial functional experiments, cells were stimulated with LPS (1 μg/mL), and endothelial barrier function and the inflammatory response were examined at various time points. For cell transfection experiments, cells were transduced with lentivirus encoding METTL3 at an MOI of 20 (BrainVTA, Wuhan, China). The TRIM59 overexpression plasmid was commercially obtained from Tsking (Beijing, China). METTL3 siRNA, YTHDF1 siRNA, Trim59 siRNA and their NCs were purchased from RiboBio (Guangzhou, China). BrainVTA. Plasmids and siRNAs were transfected with Lipofectamine 3000 (Invitrogen) according to the manufacturer’s instructions. All sequences are summarized in **Table S3**.

### 2.3 Total RNA Extraction and RT-qPCR

Total RNA was isolated from tissues and cells by using TRIzol reagent (Invitrogen Life Technologies) and quantified by measuring the absorbance at 260/280 nm using a NanoDrop One C (Thermo Fisher Scientific, USA). cDNA was prepared using HiScript III RT Supermix for qPCR (+gDNA wiper) according to the manufacturer’s instructions (Vazyme, Nanjing, China). Real-time quantitative PCR was performed using ChamQ SYBR Color qPCR Master Mix (Vazyme) in a Roche Light-Cycler 480 II real-time PCR system (Roche). Relative expression was calculated using the 2−ΔΔCT method, and the data were normalized to β-actin or 18S rRNA. The primers are listed in **Table S1**.

### 2.4 Western Blotting

Lung samples and cells were lysed using radioimmunoprecipitation assay (RIPA) buffer containing PMSF and protease inhibitors. Samples were collected, and protein concentrations were determined using a BCA protein assay kit (Pierce, USA). The proteins were subjected to 10% SDS–PAGE, transferred to a polyvinylidene fluoride (PVDF) membrane and then blocked with 5% nonfat milk. The membranes were incubated with primary antibodies overnight at 4 °C, followed by HRP-conjugated secondary antibody incubation for 1 h at room temperature. Proteins were visualized with an enhanced chemiluminescence kit (Pierce, USA) on a Chemi-Doc™ XRS^+^ system (Bio–Rad). Antibody references are listed in **Table S2**.

### 2.5 H&E Staining and Immunohistochemistry

Lung tissues were fixed with 4% paraformaldehyde and embedded in paraffin. H&E staining was performed according to the manufacturer’s instructions. Sections were also stained for METTL3, and the expression level was measured. Immunostaining was performed using the streptavidin-peroxidase immunohistochemical method. The sections were incubated overnight at 4 °C with primary antibodies and then incubated with biotin-labeled secondary antibodies at 37°C for 30 min. The sections were further developed with diaminobenzidine. Images were captured under a microscope (IX70, Olympus, Japan).

### 2.6 ELISA

The levels of inflammatory cytokines in sera and BALF from mice were examined using ELISA kits (Multi Sciences Biotech) according to the manufacturer’s instructions.

### 2.7 Quantibody Mouse Inflammation Array

A selection of 40 cytokines was analyzed with a Quantibody Mouse Inflammation Array (QAM-INF-3, RayBiotech) according to the instructions of the manufacturer. Briefly, specific capture antibodies were bound to the glass surface. After incubation with the samples, a second biotin-labeled detection antibody was added. The streptavidin-conjugated Cy3 equivalent dye was then added to the cytokine-antibody-biotin complex, and the complex was visualized by a laser scanner (InnoScan 300 Microarray Scanner).

### 2.8 TEER Measurement

HULEC-5a cells were seeded in the upper chamber of a Costar Transwell insert at a density of 1 × 10^5^ cells/cm^2^, and 600 µl of media was added to each lower chamber. Cells were incubated with 1 μg/ml LPS. Simultaneous transient TEER was measured using a MERSST×01 electrode according to the manufacturer’s instructions (EMD Millipore Corporation, Billerica MA) every two hours. TEER values were calculated by subtracting the blank value from the filter and multiplying by the surface area of the filter.

### 2.9 RNA m^6^A Modification Quantification

RNA m^6^A methylation quantification was performed using an m^6^A RNA methylation quantification kit (Abcam) according to the manufacturer’s instructions. Briefly, total RNA was isolated using TRIzol reagent (Invitrogen Life Technologies), and mRNA was purified with a Dynabeads mRNA purification kit (Thermo Scientific). Two hundred nanograms of mRNA was bound to strip wells at 37 °C for 90 min. An m^6^A capture antibody and detection antibody were added individually. The signal was then quantified by a microplate spectrophotometer at 450 nm.

### 2.10 RNA-Seq, MeRIP-seq and Data Analysis

For RNA-seq, total RNA was first extracted from HULEC-5a cells with stable METTL3 knockdown and the corresponding vector-transfected cells. The quality and quantity of the RNA were assessed by a NanoDrop™ ND-1000. mRNA extraction was performed using a TruSeq Stranded Total RNA Library Prep Kit (Illumina). RNA libraries were constructed using a QIAquick Gel Extraction Kit (Qiagen). Libraries were sequenced using Illumina HiSeq 4000 platforms.

MeRIP-Seq (m6A-Seq) was performed by Cloudseq Biotech Inc. (Shanghai, China) according to the published procedure with slight modifications. Briefly, 500 ng of fragmented mRNA was reserved as an input control for RNA-seq, and 5 μg of fragmented mRNA was incubated with 5 μg of anti-m6A polyclonal antibodies (Synaptic Systems, 202003) in IP buffer (150 mM NaCl, 0.1% NP-40, 10 mM Tris-HCl, pH 7.4) for 2 h at 4°C. The mixture was then immunoprecipitated by incubation with protein-A beads (Thermo Fisher) at 4 °C for an additional 2 h. Then, bound mRNAs were eluted from the beads and extracted with TRIzol reagent (Thermo Fisher) according to the manufacturer’s instructions. The purified mRNAs were used for RNA-seq library generation with the NEBNext Ultra™ RNA Library Prep Kit (NEB). Both the input sample (without immunoprecipitation) and the m6A IP sample were subjected to 150-bp paired-end sequencing on an Illumina HiSeq sequencer.

Paired-end reads were harvested from an Illumina HiSeq 4000 sequencer, and quality was controlled by Q30. After 3’ adaptor trimming and low-quality read removal by cutadapt software (v1.9.3), the reads were aligned to the reference genome (UCSC MM10) with Hisat2 software (v2.0.4). Methylated sites on RNAs (peaks) were identified by MACS software. Differentially methylated sites on RNAs were identified by diffReps. The peaks identified by both software programs overlapping with exons of mRNA were determined and chosen by homemade scripts.

### 2.11 Methylated RNA ImmunOprecipitation qPCR

Total RNA (500 μg) was isolated with TRIzol reagent, and poly(A)+ mRNA was purified using a Dynabeads mRNA purification kit. Then, the mRNA was denatured at 70 °C for 10 min, fragmented and immunoprecipitated with anti-m6A antibodies in 1 ml of buffer containing RNasin Plus RNase inhibitor (400 U; Promega Corporation, N2611), 50 mM Tris-HCl, pH 7.4, 750 mM NaCl, and 0.5% (vol:vol) Igepal CA-630 (Sigma–Aldrich, I8896) for 2 h at 4°C. Dynabeads Protein G (Thermo Fisher Scientific, 10003D) was washed, added to the mixture and incubated for 2 h at 4°C with rotation. m^6^A RNA was eluted twice with 6.7 mM N6-methyladenosine 5’-monophosphate sodium salt at 4 °C for 1 h and precipitated with 5 μg of glycogen and a one-tenth volume of 3 M sodium acetate in 2.5 volumes of 100% ethanol at -80 °C overnight. m^6^A enrichment was determined by RT–qPCR analysis. Fragmented mRNA was directly incubated with m^6^A antibody-containing buffer and treated similarly.

### 2.12 RNA Immunoprecipitation

The RIP assay was performed using a MagnaRIP RNA-Binding Protein Immunoprecipitation Kit (Millipore, MA, USA) according to the manufacturer’s instructions. Briefly, the corresponding cell lysates were incubated with beads coated with 5 μg of control IgG (Beyotime, Shanghai, China) or anti-YTHDF1 antibody (Abcam, MA, USA) with rotation at 4°C overnight. Next, total RNA was extracted and analyzed by RT-qPCR.

### 2.13 RNA Stability Analysis

HULEC-5a cells were cultured in six-well culture plates to 80% confluence. Actinomycin D was added at a final concentration of 5 μg/ml. Cells were collected at 0, 4 and 8 h after treatment, total RNA was extracted, RT–qPCR was performed as described above, and β-actin was used as a loading control for normalization. For each RNA transcript of interest, a semilog graph was plotted, and the RNA half-life (t(1/2)) was determined in each condition tested.

### 2.14 Statistical Analysis

GraphPad Prism software (version 8.0 for Windows, GraphPad Software Inc., La Jolla, CA) was used for statistical analyses. The band intensity in western blot images was quantified with ImageJ software. Values are expressed as the means ± SD of at least three independent experiments. The Student’s t test was used to assess the statistical significance of differences between two groups. For multiple groups, significance was evaluated by one-way ANOVA with the Bonferroni test (homogeneity of variance) or Tamhanes’s T2 test (heterogeneity of variance). The survival rate was analyzed by the log-rank test. A value of *p*<0.05 was considered statistically significant.

## 3 Results

### 3.1 Dysregulation of m^6^A Modification in Sepsis-Induced ARDS

**Figure 1:**
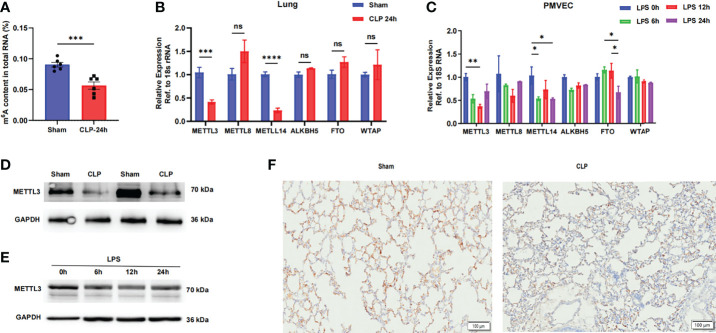
The METTL3-m^6^A pathway is downregulated in sepsis-induced ARDS. **(A)** ELISA was used to measure mRNA m^6^A levels in sham and CLP-24 h lungs. **(B, C)** RT-qPCR analysis showing the mRNA expression of m^6^A-related genes in mouse lungs and PMVECs. 18S rRNA was used as the internal control. **(D, E)** Immunoblot showing METTL3 protein expression in mouse lungs and PMVECs. GAPDH was used as the loading control. **(F)** Representative images of METTL3 immunostaining in sham lungs and CLP lungs. Scale bars, 100 mm. All data are expressed as the mean ± SD of three independent experiments (n=6). *p < 0.05; **p<0.01; ***p < 0.001; ****p < 0.0001; ns, no significance.

To elucidate the functional role of the m^6^A modification in sepsis-induced ARDS, we first evaluated m^6^A RNA levels in normal and septic mouse lungs. We found that the m^6^A levels in total RNA were significantly reduced in septic lungs *via* colorimetric ELISA (**Figure 1A**). The m^6^A modification is mainly regulated by m^6^A writers and erasers, so we hypothesized that the decreased m^6^A RNA levels in septic lungs were caused by the dysregulation of these genes. We then compared the mRNA levels of m^6^A writers (METTL3, METTL8, METTL14 and WTAP) and erasers (ALKBH5 and FTO) in lung tissues and PMVECs. The methyltransferase METTL3 and METTL14 were significantly downregulated in septic lungs and LPS-stimulated PMVECs, while the expression of the other genes was not significantly different (**Figures 1B, C**). The protein expression of METTL3 was also significantly lower in septic lung tissues than in normal lung tissues, as determined by western blotting (**Figure 1D** and **Supplementary Figure 1A**). In addition, the protein level of METTL3 was significantly decreased in LPS-treated PMVECs (**Figure 1E** and **Supplementary Figure 1B**). These results were confirmed in lung tissues with sepsis-induced ARDS, and the results showed that the expression of METTL3 was significantly decreased in septic lung tissues compared with normal lung tissues by immunohistochemistry (**Figure 1F**). Taken together, these results reveal that m^6^A modification and METTL3 levels are decreased in sepsis-induced ARDS and that the occurrence of sepsis may be related to dysregulation of the m^6^A methyltransferase METTL3.

### 3.2 METTL3 Is Associated With Aberrant m6A Modification in Sepsis-Induced ARDS *In Vivo*

**Figure 2:**
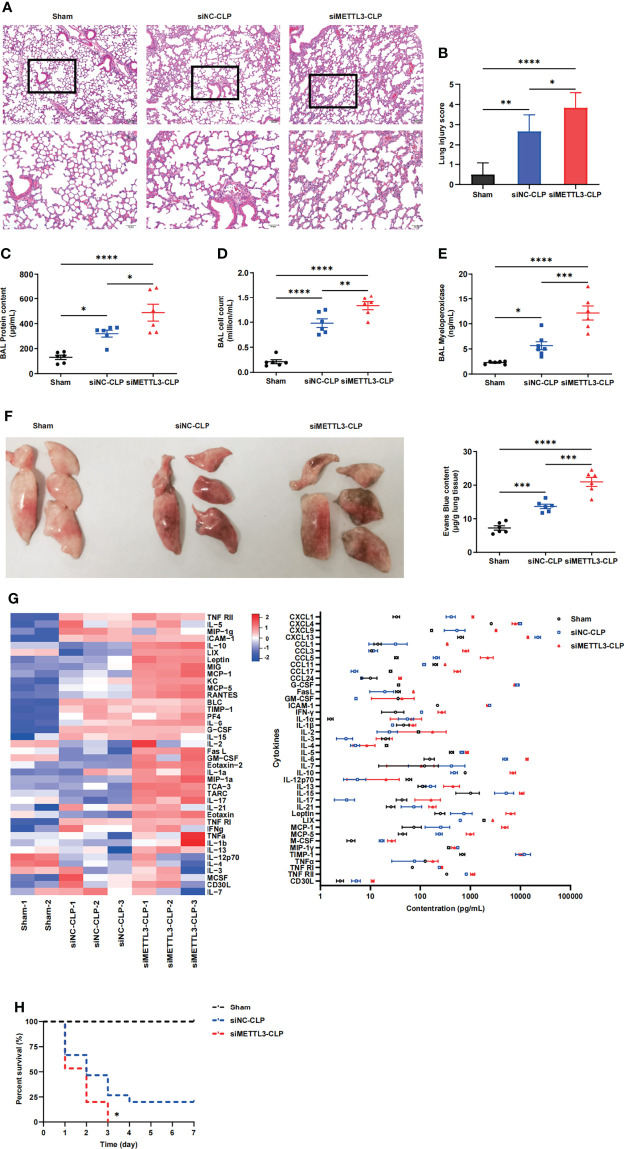
METTL3 deletion exacerbates lung endothelial injury in CLP models. **(A)** Hematoxylin and eosin (H&E)-stained lung sections from sham, CLP-NC siRNA and CLP-METTL3 siRNA mice. Scale bars, 100 μm. **(B)** Pathological lung injury scores of sham, CLP-NC siRNA and CLP-METTL3 siRNA mice. **(C–E)** Protein content **(C)**, cell counts **(D)** and MPO activity **(E)** in the BALF of CLP-METTL3 siRNA mice compared with sham and CLP-NC siRNA mice. **(F)** Whole lung images and Evans blue levels in sham, CLP-NC siRNA and CLP-METTL3 siRNA mice. **(G)** Quantibody Mouse Inflammation Array analysis of circulating blood from sham, CLP-NC siRNA and CLP-METTL3 siRNA mice. **(H)** Survival analysis of CLP-METTL3 siRNA mice compared with sham and CLP-NC siRNA mice. All data are expressed as the mean ± SD of three independent experiments (n=6 for A-G and n=15 for H). *p < 0.05; **p < 0.01; ***p < 0.001; ****p < 0.0001.

To identify the role of METTL3 in sepsis-induced ARDS, we constructed METTL3-knockdown models in mice *via* intranasal administration of METTL3 siRNA or negative control (NC) siRNA. The knockdown efficiency of METTL3 was validated at both the mRNA and protein levels (**Supplementary Figures 2A, B**). C57BL/6 mice were subjected to cecal ligation and puncture (CLP) and pretreated intranasally with METTL3 siRNA or NC siRNA 24 h before injury. We subsequently investigated the effect of METTL3 on sepsis-induced lung injury. Histologic examination of mice in the sham group revealed normal lungs that were characterized by thin alveolar walls with occasional alveolar macrophages and few neutrophils. However, the mice in the METTL3 siRNA-CLP group demonstrated significantly increased neutrophil infiltration, hemorrhage, hyaline membrane formation, and thickening of the alveolar walls compared with those in the NC siRNA-CLP group (**Figure 2A**). These observations were confirmed by lung injury score evaluations (**Figure 2B**) and suggested that METTL3 deficiency significantly exacerbated sepsis-induced lung injury. Additionally, increases in bronchoalveolar lavage fluid (BALF) protein concentrations and cell numbers were representative of augmented endothelial permeability. Correspondingly, the cell counts, protein and pro-inflammatory cytokines expression levels in BALF significantly increased after METTL3 siRNA administration compared with NC siRNA administration (**Figures 2C, D** and **Supplementary Figures 2C–E**). We also examined MPO activity to evaluate neutrophil accumulation in lung tissues. Knockdown of METTL3 significantly elevated MPO activity in septic mice (**Figure 2E**). Mice with METTL3 knockdown exhibited increased vascular leakage, as assessed by Evans blue tissue dispersion (**Figure 2F**). Furthermore, a panel of inflammatory markers was measured in circulating blood to evaluate the systemic inflammatory response after lung injury. Among the 40 inflammatory markers tested, 12 were increased at least 2-fold over those in the NC siRNA-CLP group (**Figure 2G**). Notably, siMETTL3 treatment resulted in a significant increase in CLP-induced death of the animals; specifically, the mice did not survive after 72h (**Figure 2H**). Overall, these data suggest that knockdown of METTL3 aggravates damage to endothelial barrier integrity and the inflammatory response caused by sepsis.

### 3.3 METTL3 Regulates Vascular Endothelial Barrier Function *In Vitro*

**Figure 3:**
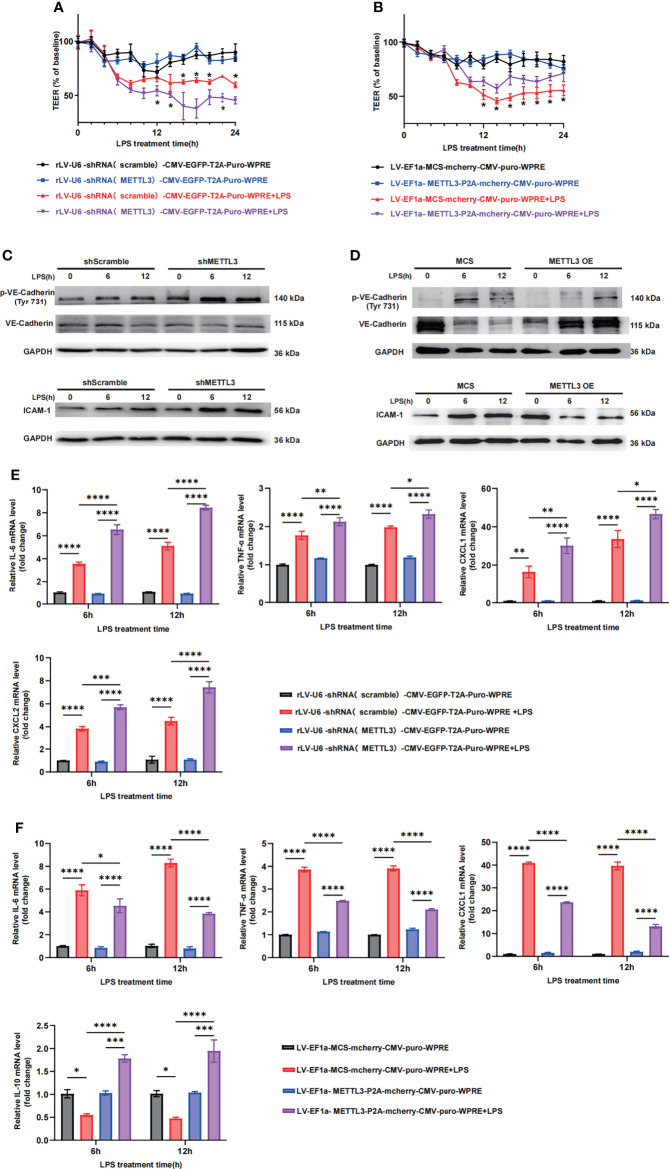
METTL3 regulates vascular endothelial barrier function *in vitro.* **(A, B)** TEER was used to measure endothelial permeability in HULEC-5a cells transfected with scramble or METTL3 shRNA **(A)** and in HULEC-5a cells with or without METTL3 overexpression **(B)**. **(C, D)** Immunoblot showing the protein expression of VE-Cadherin and ICAM-1 in HULEC-5a cells transfected with scramble or METTL3 shRNA **(C)** and in HULEC-5a cells with or without METTL3 overexpression **(D)**. **(E, F)** RT-qPCR analysis showing inflammatory cytokine mRNA expression in HULEC-5a cells transfected with scramble or METTL3 shRNA **(E)** and in HULEC-5a cells with or without METTL3 overexpression **(F)**. All data are expressed as the mean ± SD of three independent experiments. *p < 0.05; **p < 0.01; ***p < 0.001; ****p < 0.0001.

Since the expression of METTL3 was significantly downregulated in septic lungs, we further investigated its crucial role in endothelial barrier maintenance. METTL3 expression may vary in different cells, so the human lung vascular endothelial cell line HULEC-5a and primary pulmonary microvascular endothelial cells isolated from mouse lungs were chosen to establish a lung injury cell-like model. To investigate the crucial role of METTL3 in endothelial barrier maintenance, we established stable METTL3-knockdown HULEC-5a cells (**Supplementary Figure 3A**) and transfected METTL3 siRNA into PMVECs (**Supplementary Figure 3C**). Cells were stimulated with LPS at various time points. Transendothelial electrical resistance (TEER) was measured to assess barrier function. The downregulation of METTL3 induced a dramatic loss of monolayer resistance at 6 h, which was sustained for at least 24 h (**Figure 3A**), suggesting that METTL3 knockdown significantly increased the permeability of HULEC-5a cells. Consistent with these observations, we also observed loss of VE-cadherin junctions and the accumulation of ICAM-1 when HULEC-5a cells were treated with METTL3 shRNA (**Figure 3C** and **Supplementary Figure 3D**), indicating that METTL3 suppression destroyed endothelial permeability and aggravated endothelial barrier dysfunction. Furthermore, downregulation of METTL3 led to higher inflammatory cytokine expression than that induced by the shRNA controls (**Figure 3E**). Moreover, similar effects of METTL3 were confirmed, including effects on endothelial permeability and the inflammatory response, when PMVECs were treated with METTL3 siRNA (**Supplementary Figures 3E, F**).

Subsequently, we established stable METTL3-overexpressing HULEC-5a cells. METTL3 overexpression was verified by RT-qPCR and western blotting (**Supplementary Figure 3B**). As expected, METTL3 overexpression prominently improved endothelial monolayer resistance (**Figure 3B**) and elevated the expression of VE-cadherin junctions (**Figure 3D** and **Supplementary Figure 3G**). In addition, the inflammatory response was effectively inhibited (**Figure 3F**). Collectively, our *in vitro* results demonstrate the critical role of METTL3 in the regulation of vascular endothelial barrier function in sepsis-induced lung injury.

### 3.4 METTL3-Mediated m^6^A Modification of Trim59 mRNA Maintains Its YTHDF1-Dependent Stability

**Figure 4:**
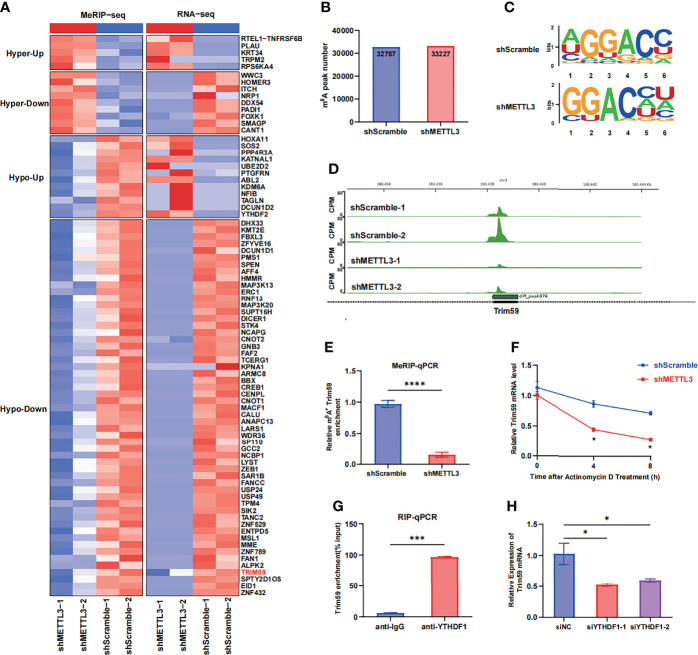
METTL3-mediated m^6^A modification of Trim59 mRNA maintains its YTHDF1-dependent stability **(A)** Heatmap showing the clusters with significant changes in both the RNA expression level (RNA-seq) and m^6^A level (MeRIP-seq) in scramble and METTL3 shRNA-treated HULEC-5a cells. **(B)** Number of m^6^A peaks in scramble and METTL3 shRNA-treated HULEC-5a cells. **(C)** Global profiling of m6A in HULEC-5a cells and the sequence motif identified from the top 1000 m^6^A peaks. **(D)** MeRIP-seq plotted the m^6^A abundances on Trim59 mRNA transcripts in HULEC-5a cells. **(E)** MeRIP-qPCR examined METTL3-mediated Trim59 mRNA m^6^A modifications. **(F)** RT-qPCR analysis showing the levels of Trim59 expression in METTL3-downregulated HULEC-5a cells treated with actinomycin D (2 μg/mL) at the indicated time points. **(G)** RNA immunoprecipitation (RIP)-qPCR assay showing the enrichment of YTHDF1 binding to Trim59 m^6^A modification sites. **(H)** RT-qPCR analysis showing the levels of Trim59 expression in YTHDF1-knockdown HULEC-5a cells. All data are expressed as the mean ± SD of three independent experiments. *p < 0.05; ***p < 0.001; ****p < 0.0001.

To identify the molecular mechanism by which METTL3 regulates endothelial barrier function, we performed RNA-seq and MeRIP-seq in HULEC-5a cells with stable METTL3 knockdown and control cells. The RNA-seq results revealed that 437 transcripts were significantly downregulated (fold change <0.5) after METTL3 knockdown. The MeRIP-seq results revealed that the m^6^A peaks in 1011 transcripts were decreased in abundance (fold change >1.2). Intriguingly, 55 transcripts overlapped in the RNA-seq and MeRIP-seq data (**Figure 4A** and **Supplementary Figure 4A**). According to our literature search, the RNA-Seq and MeRIP-seq results and Gene Ontology (GO) analysis, five genes (Tanc2, Trim59, Dhx33, Map3k13 and Map3k20) related to immune response regulation and cell adhesion were chosen (**Supplementary Figures 4C, D**). Next, we validated the mRNA levels of these five candidate genes in METTL3-knockdown HULEC-5a cells. Only Trim59 was consistently downregulated by METTL3 in HULEC-5a cells (**Supplementary Figure 4B**). In total, m^6^A sequencing identified 32,757 and 33,227 m^6^A peaks in control and m^6^A-knockdown cells, respectively (**Figure 4B**). When the m^6^A methylomes were mapped, the m6A consensus motif GGAC was identified (**Figure 4C**). A sharp decrease in the size of the m^6^A peak was identified around the exon of Trim59 mRNA in shMETTL3-treated HULEC-5a cells compared with cells in the control (**Figure 4D**). The MeRIP-seq results were validated by MeRIP-qPCR, which showed that compared with that in the shScramble group, the m^6^A-specific antibody significantly reduced the enrichment in Trim59 mRNA induced by METTL3 knockdown (**Figure 4E**). Considering that m^6^A modification positively regulates the mRNA level of Trim59, we then investigated whether m^6^A modification affected the stability of Trim59 mRNA. HULEC-5a cells were treated with the transcription inhibitor actinomycin D. RT–qPCR analysis showed that METTL3 knockdown shortened the half-life of Trim59 (**Figure 4F**). We then investigated the mechanism by which the m^6^A modification regulated the expression of Trim59. The YT521-B homology (YTH) domain-containing protein (YTHDF) family has been identified as an m^6^A reader that can influence mRNA stability (20–22). We hypothesized that Trim59 transcripts were recognized by the YTH family, the members of which act as m^6^A readers and promote the translation of the modified transcripts. RIP assays showed that YTHDF1 but not YTHDF2 or YTHDF3 was markedly enriched in Trim59 mRNA, which indicates the interaction between YTHDF1 and Trim59 mRNA (**Figure 4G**). The siRNA targeting YTHDF1 with the highest efficiency was chosen (**Supplementary Figures 4E, F**). YTHDF1 silencing decreased LPS-induced Trim59 expression in HULEC-5a cells (**Figure 4H**). Moreover, YTHDF1 overexpression partially restored the decreased level of Trim59 in METTL3-knockdown HULEC-5a cells (**Supplementary Figure 4G**). Taken together, these data indicate that METTL3-mediated m^6^A modification maintains Trim59 expression *via* YTHDF1-dependent Trim59 mRNA stability.

### 3.5 METTL3 Regulates Endothelial Function by Targeting Trim59

**Figure 5:**
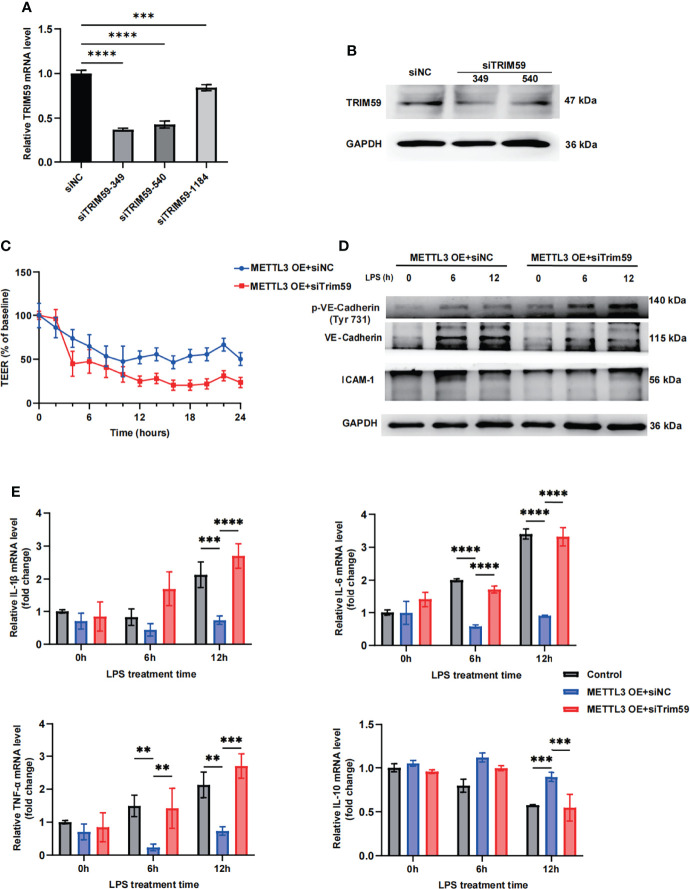
METTL3 regulates endothelial function by targeting Trim59. **(A)** RT-qPCR analysis showing the levels of Trim59 expression after siRNA transfection. **(B)** Immunoblot showing Trim59 protein expression after siRNA transfection. **(C)** TEER was used to measure endothelial permeability in METTL3-overexpressing HULEC-5a cells with or without Trim59 siRNA. **(D)** Immunoblot showing the protein expression of VE-Cadherin and ICAM-1 in METTL3-overexpressing HULEC-5a cells with or without Trim59 siRNA. **(E)** RT-qPCR analysis showing inflammatory cytokine mRNA expression in METTL3-overexpressing HULEC-5a cells with or without Trim59 siRNA. All data are expressed as the mean ± SD of three independent experiment. **p < 0.01; ***p < 0.001; ****p < 0.0001.

To further identify whether METTL3 regulates endothelial function by targeting Trim59, we cotransfected Trim59 siRNA into METTL3-overexpressing HULEC-5a cells. The gene expression inhibition efficiency is shown in **Figures 5A, B** and **Supplementary Figure 5A**. The transendothelial electrical resistance (TEER) results showed that knockdown of Trim59 in METTL3-overexpressing cells partially reversed the protective effects of METTL3 on LPS-induced endothelial injury (**Figure 5C**). Moreover, we found that METTL3 overexpression-mediated endothelial barrier recovery was significantly inhibited when Trim59 expression was downregulated (**Figure 5D** and **Supplementary Figure 5B**). In addition, knockdown of Trim59 exacerbated the endothelial inflammatory response (**Figure 5E**). Taken together, our data reveal that METTL3 ameliorates LPS-induced vascular endothelial injury by partially regulating Trim59 expression.

### 3.6 METTL3 Inhibits Endothelial Injury in Sepsis-Induced ARDS Through Trim59-Associated NF-κB Inactivation

**Figure 6:**
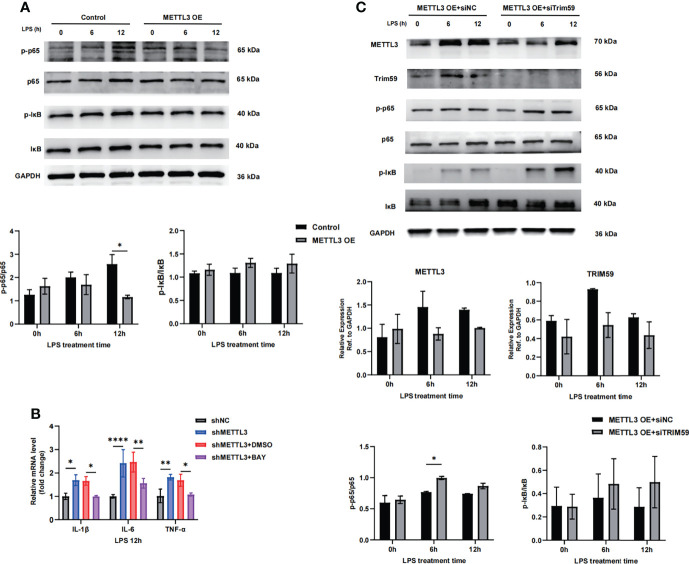
METTL3 inhibits endothelial injury in sepsis-induced ARDS through Trim59-associated NF-κB inactivation. **(A, C)** Immunoblot showing p65 and IκB phosphorylation in HULEC-5a cells treated with or without METTL3 overexpression **(A)** and METTL3-overexpressing HULEC-5a cells with or without Trim59 siRNA **(C)**. **(B)** RT-qPCR analysis showing inflammatory cytokine and endothelial functional marker mRNA expression in METTL3-overexpressing HULEC-5a cells treated with or without the NF-κB inhibitor BAY. All data are expressed as the mean ± SD of three independent experiments. *p < 0.05; **p < 0.01; ****p < 0.0001.

Trim59 participates in many pathological processes, such as inflammation, cytotoxicity and tumorigenesis, as a member of the TRIM protein superfamily. Trim59 expression is inhibited by LPS and TLR3 ligands in macrophages (23, 24). Furthermore, Trim59 interacts with evolutionarily conserved signaling intermediates in Toll pathways and negatively regulates NF-κB- and IRF-3/7-mediated signaling pathways (25). To determine whether METTL3-mediated inhibition of endothelial damage is involved in regulating Trim59, we assessed the effects of METTL3 on NF-κB pathway factor expression. As shown in **Figure 6A**, METTL3 overexpression significantly inhibited the NF-κB pathway by decreasing p65 phosphorylation, while inhibiting p65 phosphorylation with the specific NF-kB signaling inhibitor BAY decreased the expression of endothelial injury markers induced by METTL3 knockdown (**Figure 6B**). In addition, we cotransfected Trim59 siRNA into METTL3-overexpressing HULEC-5a cells and observed that the downregulation of Trim59 significantly promoted the activation of the NF-κB pathway (**Figure 6C**). These data suggest that METTL3 regulates endothelial function in sepsis-induced ARDS through Trim59-associated NF-κB inactivation.

## 4 Discussion

m^6^A is a ubiquitous RNA epigenetic modification in eukaryotes that is dynamically and reversibly regulated by methyltransferases and demethylases and is recognized by m^6^A binding proteins to exert biological functions. Emerging evidence strongly suggests that m^6^A modification is a key regulator of inflammation, innate immunity and tumorigenesis (26, 27). In this study, we demonstrate a novel function of the m^6^A modification in sepsis-induced ARDS. We discovered that METTL3-mediated m^6^A modification of mRNA was dysregulated in septic lungs. In addition, our study showed the critical role of METTL3 in the regulation of vascular endothelial barrier function. Mechanistically, METTL3 inhibits endothelial injury in sepsis-induced ARDS through Trim59-associated NF-κB inactivation. These data provide insight for the development of a novel therapeutic strategy for sepsis-associated ARDS treatment.

METTL3 is a key methyltransferase associated with m^6^A modification and directly promotes mRNA translation. Recent studies have revealed that METTL3 is involved in various biological processes, including the cell cycle, cell proliferation and differentiation, and the inflammatory response. These biological processes are closely associated with several diseases, such as cancer, inflammatory diseases, immunological diseases, and metabolic diseases (28–30). Several studies have shown that METTL3 promotes the inflammatory response by weakening the malabsorptive activity of LCFAs *in vitro (* 31). In addition, knocking down METTL3 inhibits the IL-1β-induced inflammatory response and extracellular matrix (ECM) synthesis (32, 33). In contrast, METTL3 knockdown inhibits osteoblast differentiation and Smad-dependent signaling and activated the inflammatory response by regulating MAPK signaling in LPS-induced inflammation (34). Moreover, METTL3 inhibited apoptosis and autophagy in chondrocytes by mediating Bcl2 stability *via* m^6^A modification (35, 36). A recent study showed that hepatocyte-specific deletion of Mettl3 drives NAFL-to-NASH progression by increasing CD36-mediated hepatic free fatty acid uptake and CCL2-induced inflammation (37). However, it is unclear whether METTL3 promotes or inhibits inflammation in sepsis-induced lung injury. We showed that METTL3 levels were decreased in sepsis-induced ARDS, and the knockdown of METTL3 exacerbated the damage to endothelial barrier integrity and the inflammatory response caused by sepsis in mice. Furthermore, *in vitro* studies indicated that METTL3 could regulate vascular endothelial barrier function in LPS-induced HULEC-5a cells.

Integrative RNA-seq and MeRIP-seq analyses identified Trim59, a member of the TRIM family, as the top repressed gene in METTL3-knockdown HULEC-5a cells. TRIM proteins are involved in the innate immune response, and recent studies have confirmed that TRIM family proteins can restrict retroviral infections and influence various signaling pathways, such as the IFN signaling and TLR signaling pathways (38–40). Additionally, TRIM family proteins participate in the regulation of cytokine gene transcription, and TRIM59 is upregulated in various cancers and promotes the development of tumors (41–43). TRIM59 in myeloid-derived macrophages protected mice from sepsis by regulating inflammation and phagocytosis and could directly regulate inflammation *via* the NF-κB signaling pathway (44), but the specific binding site or molecules involved remain unknown. In our study, we revealed that YTHDF1 could tightly bind to Trim59 mRNA. YTHDF1 silencing decreased the mRNA expression of Trim59. RIP assays further verified that YTHDF1 could enrich Trim59 mRNA, suggesting that METTL3-mediated m6A modification of Trim59 mRNA maintains its YTHDF1-dependent stability. NF-kB is well recognized as a key proinflammatory transcription factor, and the activity of the NF-kB signaling pathway promotes the development and progression of sepsis-induced endothelial damage. Our data showed that cotransfection of Trim59 siRNA into METTL3-overexpressing HULEC-5a cells significantly promoted activation of the NF-κB pathway. Therefore, we concluded that METTL3 inhibits endothelial injury in sepsis-induced ARDS through Trim59-associated NF-κB inactivation.

This study has some potential limitations. First, a modified METTL3 siRNA was used for *in vivo* validation, and a genetic METTL3 knockout and/or transgene mouse study could strengthen the conclusions. Second, it is possible that multiple functional targets of the m^6^A modification mediated by METTL3 modulate endothelial barrier function. We identified Trim59 as a major candidate by RNA-seq and MeRIP-seq analyses. However, the overall results from comprehensive knockdown/overexpression studies *in vitro* and *in vivo* support our findings.

In summary, our study provides evidence that METTL3 depletion-mediated reductions in m^6^A modification affect endothelial barrier function in sepsis-induced ARDS, which ultimately increases endothelial permeability and aggravates the inflammatory response, thereby increasing sepsis-associated mortality. These findings revealed the application value of this modification in the diagnosis, prognosis, and molecular-targeted therapy of sepsis-associated lung injury.

## Data Availability Statement

The datasets presented in this study can be found in online repositories. The name of the repository and accession number(s) can be found below: NCBI (https://www.ncbi.nlm.nih.gov/); GSE200648 and GSE200649.

## Ethics Statement

The animal study was reviewed and approved by Institutional Animal Care and Use Committee at the Renji Hospital, Shanghai Jiaotong University School of Medicine.

## Author Contributions

WY conceptualized the study. YC, YW and LZ contributed equally. The study was designed by YC and supervised by WY The manuscript was written by YC, YW and YJ provided important biological samples or research tools and provided important ideas and edited the manuscript. The other experiments were performed by CC, DT and SX. All authors have read and approved the manuscript.

## Funding

This work was supported by the National Natural Science Foundation of China(81701940 to YC, 32030043 to WY), National Key R&D Program (2018YFA0108204 to WY), Shanghai Engineering Research Center of Peri-operative Organ Support and Function Preservation (20DZ2254200 to WY), Area Municipal Commission of Health and Family Planning Funding (PWZxq2017-06 to WY), Shanghai Municipal Key Clinical Specialty (Shslczdzk03601 to WY), Shanghai Municipal Education Commission (2019Technology education-01-8 to WY).

## Conflict of Interest

The authors declare that the research was conducted in the absence of any commercial or financial relationships that could be construed as a potential conflict of interest.

## Publisher’s Note

All claims expressed in this article are solely those of the authors and do not necessarily represent those of their affiliated organizations, or those of the publisher, the editors and the reviewers. Any product that may be evaluated in this article, or claim that may be made by its manufacturer, is not guaranteed or endorsed by the publisher.
