# Supplementary material for: METTL3-Mediated N6-Methyladenosine Modification of Trim59 mRNA Protects Against Sepsis-Induced Acute Respiratory Distress Syndrome
Source: Front Immunol. 2022 May 25;13:897487. doi: 10.3389/fimmu.2022.897487 (PMC9174697; doi:10.3389/fimmu.2022.897487)
Supplement: Supplementary file 1 [file DataSheet_1.docx]

Supplementary Material

**1. Supplementary figure**

**1.1 Additional file 1: Supplementary Fig. S1**

**1.2 Additional file 2: Supplementary Fig. S2**

**1.3 Additional file 3: Supplementary Fig. S3**

**1.4 Additional file 4: Supplementary Fig. S4**

**1.5 Additional file 5: Supplementary Fig. S5**

**1.6 Additional file 6: Supplementary Fig. S6**

**2. Supplementary table**

**2.1 Additional file 7: Supplementary Table S1**

**2.2 Additional file 8: Supplementary Table. S2**

**2.3 Additional file 9: Supplementary Table. S3**

1. **Supplementary figures**

**
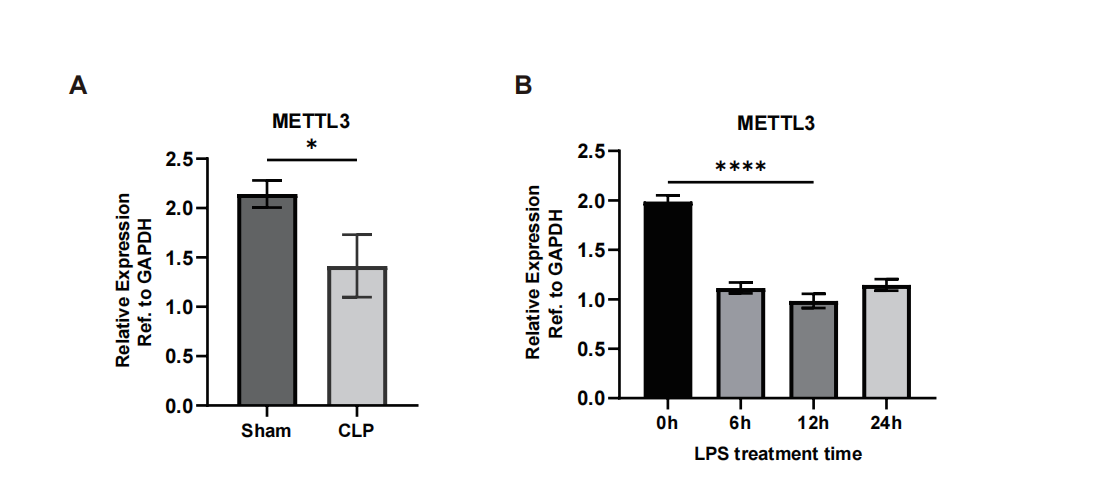
**

**Supplementary Figure 1. The METTL3-m^6^A pathway is downregulated in sepsis-induced ARDS. (Related to Figure 1)**

1. Quantification of the METTL3 protein expression level in mouse lungs. GAPDH was used as the loading control.
2. Quantification of the METTL3 protein expression level in LPS-stimulated PMVECs. GAPDH was used as the loading control.

**
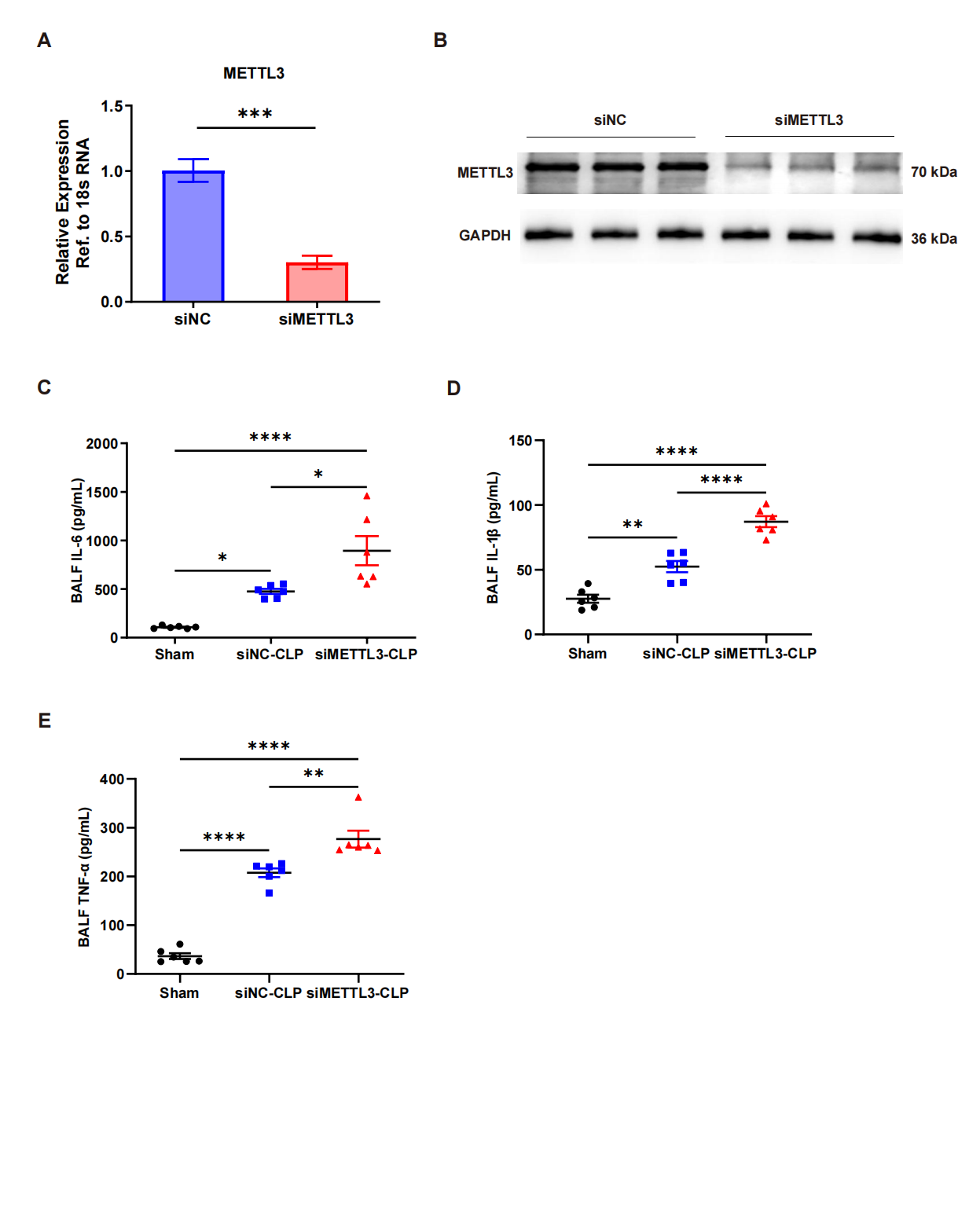
Figure S1: Supplementary Figure 2. METTL3 deletion exacerbates lung endothelial injury in CLP models. (Related to Figure 2)**

(A) qRT–PCR analysis showing METTL3 mRNA expression in mouse lungs with or without METTL3 siRNA administration. 18S rRNA was used as the internal control.

(B) Immunoblot showing METTL3 protein expression in mouse lungs with or without METTL3 siRNA administration. GAPDH was used as the loading control.

(C-E) ELISA was used to measure the levels of the inflammatory cytokines IL-6 (C), IL-1β (D) and TNF-α (E) in BALF from CLP-METTL3 siRNA mice compared with sham and CLP-NC siRNA mice.

**
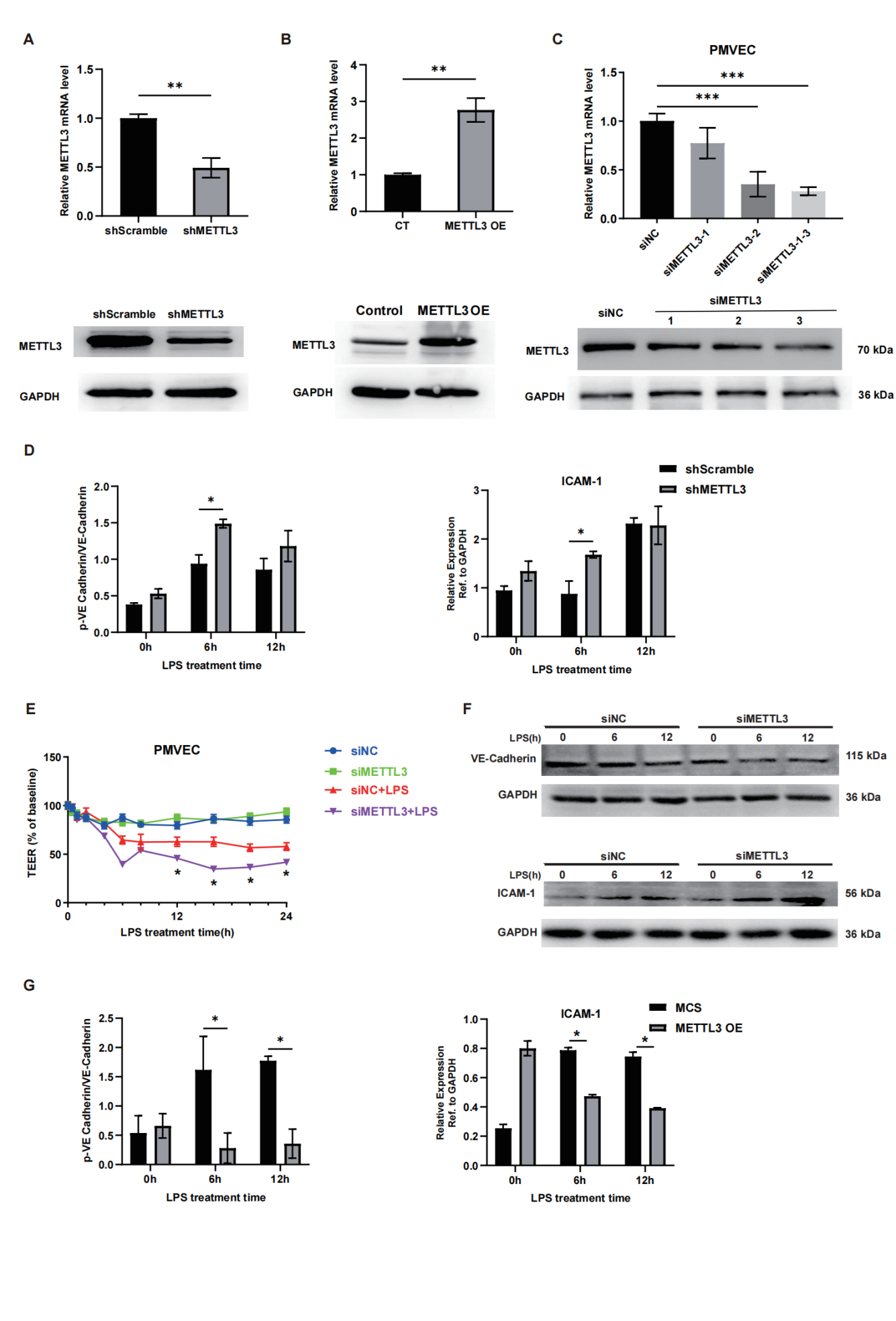
**

**Supplementary Figure 3. METTL3 regulates vascular endothelial barrier function in vitro*.* (Related to Figure 3)**

(A) METTL3 knockdown was verified by qRT–PCR (upper panel) and western blotting (lower panel) in HULEC-5a cells transfected with lentiviral vectors (containing scramble shRNA or METTL3 shRNA).

(B) METTL3 overexpression was verified by qRT–PCR (upper panel) and western blotting (lower panel) in HULEC-5a cells transfected with lentiviral vectors (containing control cDNA or METTL3 cDNA).

(C) METTL3 knockdown was verified by qRT–PCR (upper panel) and western blotting (lower panel) in PMVECs transfected with NC or METTL3 siRNA.

(D) Quantification of the phospho-VE-Cadherin/VE-Cadherin and ICAM-1 protein expression level in HULEC-5a with or without METTL3 knockdown. GAPDH was used as the loading control.

(E) TEER was used to measure endothelial permeability in PMVECs with or without METTL3 siRNA.

(F) Immunoblot showing the protein expression of VE-Cadherin and ICAM-1 in PMVECs with or without METTL3 siRNA.

(G) Quantification of the phospho-VE-Cadherin/VE-Cadherin and ICAM-1 protein expression level in HULEC-5a with or without METTL3 overexpression. GAPDH was used as the loading control.

**
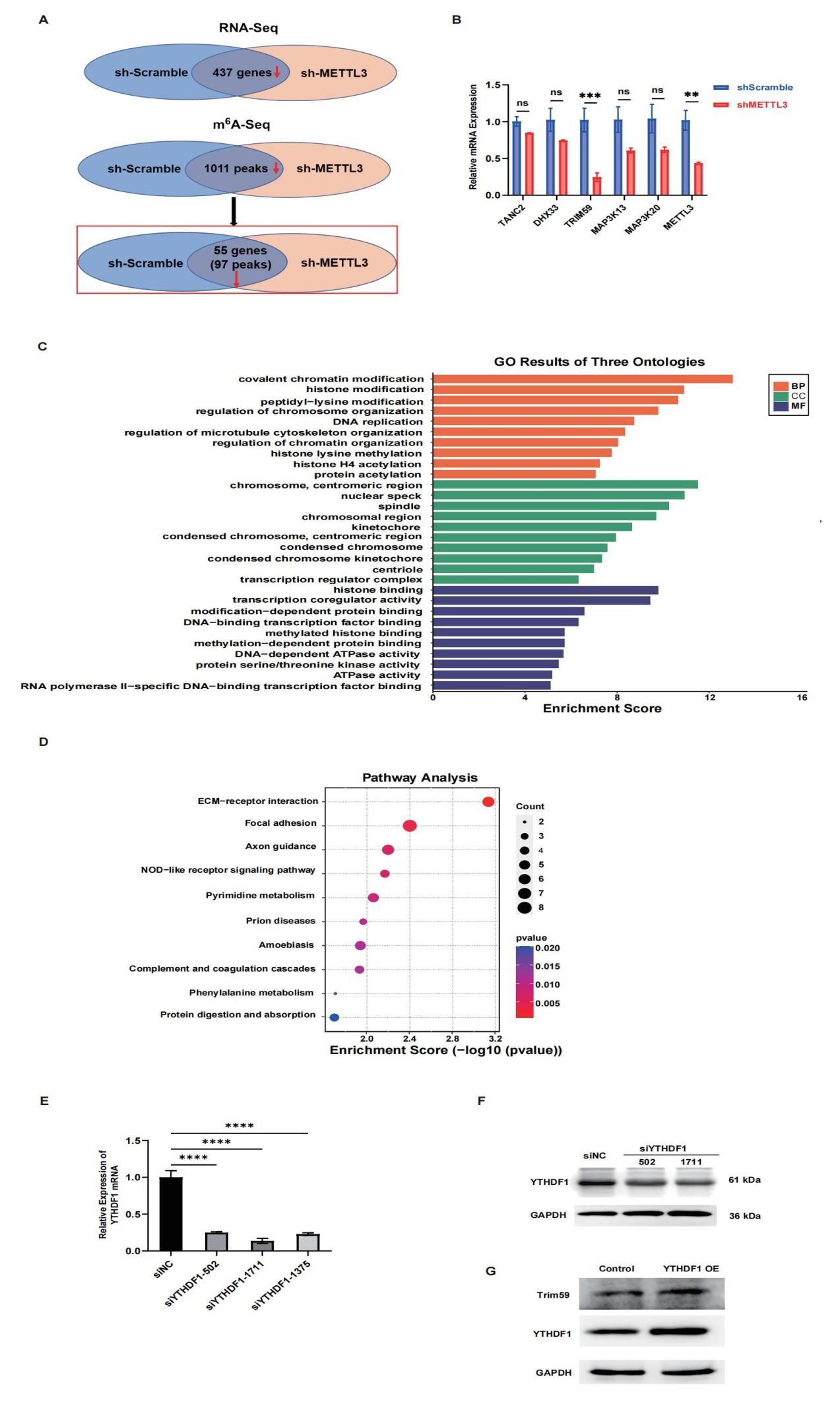
Figure S3: Supplementary Figure 4. METTL3-mediated m^6^A modification of Trim59 mRNA maintains its YTHDF1-dependent stability (Related to Figure 4).**

(A) Gene set overlap analysis showing overlaps between gene sets that were aberrant for m^6^A targets (MeRIP-seq) and gene sets for transcripts that were downregulated after METTL3 depletion (RNA-seq).

(B) qRT–PCR analysis showing candidate target mRNA expression in HULEC-5a cells transfected with lentiviral vectors (containing scramble shRNA or METTL3 shRNA).

(C) GO analysis showing the downregulated m^6^A-modified mRNA transcripts after METTL3 knockdown.

(D) KEGG pathway analysis showing the pathway associated with METTL3 downregulation.

(E) qRT–PCR analysis showing YTHDF1 mRNA expression in HULEC-5a cells with or without YTHDF1 siRNA administration. 18S rRNA was used as the internal control.

(F) Immunoblot showing YTHDF1 protein expression in HULEC-5a cells with or without YTHDF1 siRNA administration. GAPDH was used as the loading control.

(G) Immunoblot showing Trim59 protein expression in HULEC-5a cells with YTHDF1 overexpression plasmid transfection. GAPDH was used as the loading control.


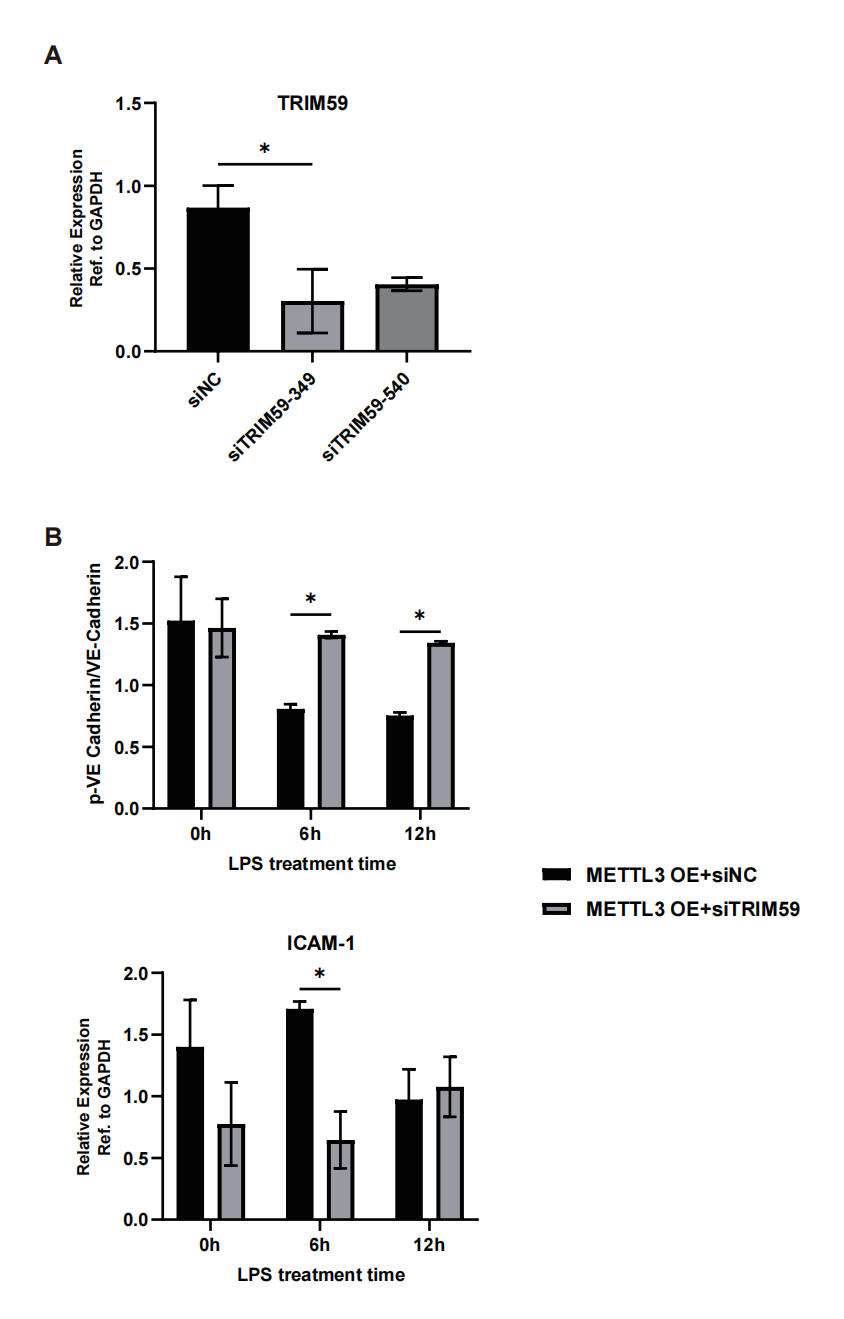


**Supplementary Figure 5. METTL3 regulates endothelial function by targeting Trim59. (Related to Figure 5)**

(A) Quantification of Trim59 protein expression level in HULEC-5a transfected with NC or Trim59 siRNA. GAPDH was used as the loading control.

(B) Quantification of the phospho-VE-Cadherin/VE-Cadherin and ICAM-1 protein expression level in METTL3-overexpressing HULEC-5a cells with or without Trim59 siRNA. GAPDH was used as the loading control.

**
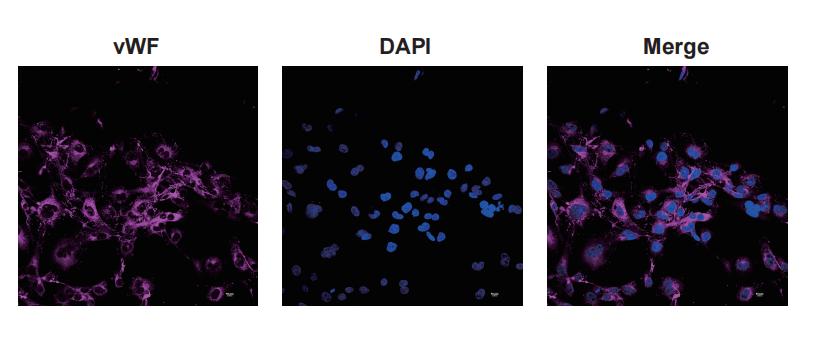
Supplementary Figure 6. Morphology and identification of primary PMVECs.** Primary cultured PMVECs were stained with Von Willebrand Factor (purple), DAPI was used to detect nuclei (blue). Scale bars, 100μm

1. **Supplementray tables**

**Table S1 Primer List**

| **Gene** | **Forward primer** | **Reverse Primer** |
| --- | --- | --- |
| Mus-VE-Cadhrein | CCACTGCTTTGGGAGCCTT | GGCAGGTAGCATGTTGGGG |
| Mus-VCAM-1 | TTGGGAGCCTCAACGGTACT | GCAATCGTTTTGTATTCAGGGGA |
| Mus-IL-1β | GAAATGCCACCTTTTGACAGTG | TGGATGCTCTCATCAGGACAG |
| Mus-TNFα | CAGGCGGTGCCTATGTCTC | CGATCACCCCGAAGTTCAGTAG |
| Mus-IL-6 | CTGCAAGAGACTTCCATCCAG | AGTGGTATAGACAGGTCTGTTGG |
| Mus-IL-10 | CTTACTGACTGGCATGAGGATCA | GCAGCTCTAGGAGCATGTGG |
| Mus-METTL3 | \| CAAGCTGCACTTCAGACGAA \| \| --- \| | GCTTGGCGTGTGGTCTTT |
| Mus-METTL8 | CCACCCAGGAAGAGTCTCAG | GGTTACACCCATGGTCAGGA |
| Mus-METTL14 | AGAAACTTGCAGGGCTTCCT | TCTTCTTCATATGGCAAATTTTCTT |
| Mus-ALKBH5 | CCCGAGGGCTTCGTCAACA | CGACACCCGAATAGGCTTGA |
| Mus-FTO | TGGGTTCATCCTACAACGG | CCTCTTCAGGGCCTTCAC |
| Mus-WTAP | GGCGAAGTGTCGAATGCT | CCAACTGCTGGCGTGTCT |
| Mus-18S rRNA | AGGGGAGAGCGGGTAAGAGA | GGACAGGACTAGGCGGAACA |
| Homo-VE-Cadhrein | AGAGTGGAGCCTGGTCTTACA | CCTTTGCTGACAATAAGCACTGG |
| Homo-ICAM1 | ATGCCCAGACATCTGTGTCC | GGGGTCTCTATGCCCAACAA |
| Homo-TRIM59 | CAATGCCAGTTGGAGCAATTTC | AAGATCCTCGTGTACTGCCAT |
| Homo-18S rRNA | CAGCCACCCGAGATTGAGCA | TAGTAGCGACGGGCGGTGTG |
| Homo-METTL3 | CATTGCCCACTGATGCTGTG | AGGCTTTCTACCCCATCTTGA |
| Homo-β-Actin | TGACGTGGACATCCGCAAAG | CTGGAAGGTGGACAGCGAGG |
| Homo-YTHDF1 | ACCTGTCCAGCTATTACCCG | TGGTGAGGTATGGAATCGGAG |
| Homo-TNFα | TCCTGCTGCACTTTGGAGTGA | TCGAGAAGATGATCTGACTGCC |
| Homo-IL-1β | CCACAGACCTTCCAGGAGAATG | GTGCAGTTCAGTGATCGTACAGG |
| Homo-IL-6 | AGACAGCCACTCACCTCTTCAG | TTCTGCCAGTGCCTCTTTGCTG |
| Homo-IL-10 | GACTTTAAGGGTTACCTGGGTTG | TCACATGCGCCTTGATGTCTG |
| Homo-CXCL1 | TGCTGCCACTAATGCTGATGT | CTCAGGAACCAATCTTTGCACT |

| **Description** | **Supplier** | **Cat No.** |
| --- | --- | --- |
| m^6^A antibody | Synaptic Systems | 202003 |
| METTL3 antibody | Proteintech | [15073-1-AP](https://www.labome.com/product/Proteintech-Group/15073-1-AP.html) |
| GAPDH antibody | Abcam | ab8245 |
| β-Tubulin antibody | Cell Signaling Technology | #2128 |
| ICAM-1 antibody | Abclonal | A5597 |
| VE-Cadherin antibody | Abcam | ab33168 |
| Phospho-VE-cadherin (Tyr731) Antibody | Invitrogen™ | 44-1145G |
| Trim59 antibody | Abcam | ab69639 |
| YTHDF1 antibody | Proteintech | 17479-1-AP |
| NF-kB p65 (phospho S536) antibody | Abcam | ab86299 |
| NF-kB p65 antibody | Abcam | ab16502 |
| Phospho-IκBα (Ser32) (14D4) antibody | Cell Signaling Technology | #2859 |
| IκBα (44D4) antibody | Cell Signaling Technology | #4812 |
| IgG Kappa Light Chain antibody | Proteintech | 14678-1-AP |
| Anti-mouse IgG, HRP-linked Antibody | Cell Signaling Technology | #7076 |
| Anti-rabbit IgG, HRP-linked Antibody | Cell Signaling Technology | #7074 |
| Evans blue | Sigma Aldrich | E2129 |
| [m^6^A RNA Methylation Assay Kit (Colorimetric)](https://www.abcam.cn/m6a-rna-methylation-assay-kit-colorimetric-ab185912.html) | Abcam | ab185912 |
| Lipopolysaccharides from Escherichia coli O111:B4 | Sigma Aldrich | L4391 |
| Mouse IL-1β ELISA Kit | Multi Sciences | 70-EK201B/3 |
| Mouse IL-6 ELISA Kit | Multi Sciences | 70-EK206/3 |
| Mouse TNF-α ELISA Kit | Multi Sciences | 70-EK282/4 |
| Mouse myeloperoxidase/MPO ELISA Kit | Multi Sciences | 70-EK2133/2 |
| Mouse Infalmmation Array Q1 | RayBiotech | QAM-INF-1 |
| RNA EntransterTM-in vivo | Engreen | 18668-11-1 |
| Lipofectamine™ 3000 Transfection Reagent | Invitrogen™ | L3000015 |
| Actinomycin D | Sigma Aldrich | SBR00013 |
| BAY 11-7082 | Target Mol | T1902 |

**Table S2 Antibody and reagent information**

**Table S3 Gene sequences information**

| Description | Target Sequences | Supplier |
| --- | --- | --- |
| Homo Trim59 siRNA-349 | GCAUUGAAUCUUUACCUGUUATT | Sangon Biotech |
| Homo Trim59 siRNA-1184 | UUUCAACCAACACAUCAUAACTT | Sangon Biotech |
| Homo Trim59 siRNA-540 | GACCUUCAAAGUGCCUAUUUGTT | Sangon Biotech |
| Homo YTHDF1 siRNA-1375 | CGAAAGAGUUUGAGUGGAATT | Sangon Biotech |
| Homo YTHDF1 siRNA-1711 | ACAUCAGGCUGGAGAAUAATT | Sangon Biotech |
| Homo YTHDF1 siRNA-502 | CCUACGGACAGCUCAGUAATT | Sangon Biotech |
| Mus METTL3 siRNA-001 | TCGGACACGTGGAGCTCTA | RiboBio |
| Mus METTL3 siRNA-002 | CTGGACGTCAGTATCTTGG | RiboBio |
| Mus METTL3 siRNA-003 | CCACTCAAGATGGGGTAGA | RiboBio |
| rLV-U6-shRNA(scramble)-CMV-EGFP-T2A-Puro-WPRE | CGAGGGCGACTTAACCTTAGG | BrainVTA |
| rLV-U6-shRNA(METTL3)-CMV-EGFP-T2A-Puro-WPRE | GCTAAACCTGAAGAGTGATAT | BrainVTA |
| LV-EF1a-MCS-mcherry-CMV-puro-WPRE |  | BrainVTA |
| LV-EF1a-METTL3-P2A-mcherry-CMV-puro-WPRE |  | BrainVTA |
| pcDNA3.1(+)-Trim59- Myc-HisA plasmid |  | Tsingke |
| pcDNA3.1(+)-YTHDF1-EYFP-Myc-HisA plasmid |  | Tsingke |
